# Nuclear medicine training and practice in Poland

**DOI:** 10.1007/s00259-014-2853-0

**Published:** 2014-08-05

**Authors:** Anna Teresińska, Bożena Birkenfeld, Leszek Królicki, Mirosław Dziuk

**Affiliations:** 1 Department of Nuclear Medicine, Institute of Cardiology, Alpejska 42, 04-628 Warsaw, Poland; 2 Department of Nuclear Medicine, Pomeranian Medical University, Unii Lubelskiej 1, 71-252 Szczecin, Poland; 3 Department of Nuclear Medicine, Warsaw Medical University, Banacha 1 A, 02-097 Warsaw, Poland; 4 Department of Nuclear Medicine, Military Institute of Medicine, Szaserów 128, 04-141 Warsaw, Poland

**Keywords:** Poland, Polish Society of Nuclear Medicine, Nuclear medicine specialization, Medical physics specialization, Radiopharmacy specialization, Nuclear medicine specialization for nurses

## Abstract

In Poland, nuclear medicine (NM) has been an independent specialty since 1988. At the end of 2013, the syllabus for postgraduate specialization in NM has been modified to be in close accordance with the syllabus approved by the European Union of Medical Specialists and is expected to be enforced before the end of 2014. The National Consultant in Nuclear Medicine is responsible for the specialization program in NM. The Medical Center of Postgraduate Training is the administrative body which accepts the specialization programs, supervises the training, organizes the examinations, and awards the specialist title. Specialization in NM for physicians lasts for five years. It consists of 36 months of training in a native nuclear medicine department, 12 months of internship in radiology, 3 months in cardiology, 3 months in endocrinology, 3 months in oncology, and 3 months in two other departments of NM. If a NM trainee is a specialist of a clinical discipline and/or is after a long residency in NM departments, the specialization in NM can be shortened to three years. During the training, there are obligatory courses to be attended which include the elements of anatomy imaging in USG, CT, and MR. Currently, there are about 170 active NM specialists working for 38.5 million inhabitants in Poland. For other professionals working in NM departments, it is possible to get the title of a medical physics specialist after completing 3.5 years of training (for those with a master's in physics, technical physics or biomedical engineering) or the title of a radiopharmacy specialist after completing 3 years of training (for those with a master's in chemistry or biology). At present, the specialization program in NM for nurses is being developed by the Medical Centre of Postgraduate Education. Continuing education and professional development are obligatory for all physicians and governed by the Polish Medical Chamber. The Polish Society of Nuclear Medicine (PTMN) organizes regular postgraduate training for physicians working in NM. Educational programs are comprehensive, covering both diagnostics and current forms of radioisotope therapy. They are aimed not only at physicians specialized/specializing in NM, but also at other medical professionals employed in radionuclide departments as well as physicians of other specialties.

## Historical notes

The first iodine treatment of thyroid disease was performed in Poznań, Poland in 1952, and the first radioisotope center in Poland was organized by Professor Maciej Gembicki at the Department of Internal Diseases at Poznań University of Medical Sciences as early as 1955–1956. In 1970, the Department was appointed by IAEA to organize international training programs in radioimmunoassay methods. In the conduct of these courses, 47 medical professionals from all over the world were trained.

In 1974, the first planar gamma camera was installed in Poland; in 1982, the first SPECT; in 2003, the first PET/CT; and in 2013, the first PET/MRI.

The Polish Society of Nuclear Medicine (PTMN, http://www.ptmn.pl) was created in 1982 from the section of nuclear medicine which had been established 10 years earlier at the Polish Society of Radiology. The first congress of PTMN, held every two years since then, took place in 1986. The Society confederates around 450 members. The Chairman of PTMN for the last two terms of office has been Prof. Leszek Królicki.

In 1987, the official journal of PTMN, ‘Problemy Medycyny Nuklearnej’ (‘Problems of Nuclear Medicine’), was launched. The international ‘Nuclear Medicine Review,’ another biannual periodical of a scientific and educational profile written in English, was established in Poland in 1998. The founders and first editors of the journal were Prof. Piotr Lass and Prof. Julian Liniecki. Since 2011, both journals have been merged and ‘Nuclear Medicine Review’ has become the official journal of PTMN (http://www.nmr.viamedica.pl). The periodical is indexed at Index Copernicus, Scopus, EMBASE, Index Medicus/Medline and is an official journal of the Polish, Hungarian, and Serbian Societies of Nuclear Medicine.

The Polish Society of Nuclear Medicine is one of the founding members of EANM, established in 1994.

## Organizational workforce

At present, there are 63 departments (55 public and 8 non-public) performing conventional imaging nuclear medicine in Poland and there are 103 operating gamma cameras (16 SPECT/CT, 56 SPECT, 31 planar cameras). The majority of applied radiopharmaceuticals are produced by a Polish radioisotope center (POLATOM).

After 10 years since the first PET center in Poland was created [1], there are now 24 PET/CT (12 public and 12 non-public) scanners and 1 PET/MR scanner (public) active, that is, 1 scanner per 1.5 million inhabitants. At present, the number of working cyclotrons dedicated to PET-radiopharmaceutical synthesis equals 6. Radiopharmaceuticals produced are generally used internally, as only one center produces F-18-FDG commercially and the sites not possessing a cyclotron mainly import F-18-FDG from Germany or Austria.

In both public and non-public departments, on average, about 80 % of studies are provided on the basis of concluded contracts with the National Health Fund (NFZ).

About 170,000 classic nuclear medicine studies are conducted yearly, and translate to 4.4 studies/1,000 inhabitants/year and to around 1,650 scans/camera/year. In addition, more than 20,000 therapies, designed mainly for non-cancer diseases (85 %), are performed per year.

Around 38,000 PET studies are performed in total, and these translate to about 1 study/1,000 inhabitants/year and 1,600 PET/CT scans/scanner/year.

The staff employed in nuclear medicine consists of over 1,000 persons: physicians (26 %), other personnel with university degrees (18 %), nurses (21 %), technologists (15 %), and other personnel (20 %). More than half of the doctors are represented by nuclear medicine specialists (170). The number of staff members involved in clinical PET/CT varies across the centers; more technologists than physicists or medical doctors are involved. There is a need to staff the PET/CT systems with both a radiology-trained technologist and a nuclear medicine-trained nurse. Systems with high throughput may require the availability of a larger number of technologists to limit the total radiation burden of each. There are more nuclear medicine specialists involved than radiologists, as reflected in the low frequency of contrast-enhanced PET/CT examinations; however, all sites report the engagement of radiologists. Only three sites employ dual-board-certified physicians.

The main responsibility of the National Consultant in Nuclear Medicine (Prof. Leszek Królicki, since 2000) and regional consultants is to supervise professional development in nuclear medicine and to control healthcare providers who conduct postgraduate education and residency programs in this field. They are also obliged to monitor healthcare accessibility; to control medical equipment used by healthcare providers; and to notify administration, healthcare providers, regional departments of the National Health Fund, and the Commissioner for Patients’ Rights of any abnormalities. The consultants are often members of boards and committees dealing with health policy implementation. They provide opinions on workforce needs in the field as well as ensure the implementation of current guidelines and procedures regarding diagnosis and treatment. Finally, they put forward annual reports on the task status in the field of nuclear medicine. The National Consultant is additionally obliged to advise on the tasks performed by medical examination and education centers as well as on the goals set by the National Health Program and other health policy programs. The National Consultant is also to project healthcare needs in the field of nuclear medicine, and when asked by the Health Minister, to give an opinion on the drafts of legal acts. Furthermore, the National Consultant evaluates the applications concerning healthcare provision abroad and advises the Health Minister for the appointment of a new regional consultant.

## Training resources and organization

### Nuclear medicine specialization for physicians

In Poland, nuclear medicine has been an independent specialty since 1988. At the end of 2013, the syllabus for postgraduate specialization in nuclear medicine has been modified to be in close accordance with the syllabus approved by the European Union of Medical Specialists (UEMS) [2] and is expected to be enforced before the end of 2014. The National Consultant in Nuclear Medicine is responsible for the specialization program, and the Medical Center of Postgraduate Training is the administrative body which accepts the specialization program, supervises the training, acknowledges departments where training can be done, organizes the examinations (twice a year), and finally, awards the specialist title.

Specialization in nuclear medicine lasts for five years. It consists of 36 months of training in a native nuclear medicine department, 12 months of internship in radiology, three months in cardiology, three months in endocrinology, three months in oncology, and three months in two other departments of nuclear medicine (with other technologies and/or other types of medical examinations performed). If a nuclear medicine trainee is a specialist of a clinical discipline and/or is after a long residency in Polish or foreign nuclear medicine departments, the specialization in nuclear medicine can be shortened to three years. During the training, there are obligatory courses to be attended. The basic courses include introduction to nuclear medicine (30 h) as well as radiation protection, emergency medicine, public health, and medical law. The advanced courses cover the radionuclide studies of the cardiovascular system; PET, SPECT, and hybrid imaging in oncology; diagnostics of the nervous system; radioisotope therapy; and the elements of anatomy imaging in USG, CT, and MR. After completing the training, there is a centrally organized specialization examination, which consists of a written part (test) and a practical part.

Currently, there are about 170 active nuclear medicine specialists working for 38.5 million inhabitants in Poland.

### Specializations for other professionals working in nuclear medicine departments

For those with a master's in physics, technical physics or biomedical engineering, it is possible to get the title of a medical physics specialist after completing 3.5 years of training and after passing the final national examination. The training includes 520 h of course activities (lectures, presentations, exercises) and 22 weeks of practical training. Courses on diagnostic imaging cover 60 h, and on nuclear medicine, 50 h (35 for diagnostics and 15 for therapy). Practical training contains diagnostic imaging (4 weeks) and nuclear medicine diagnostics and therapy (4 weeks); training in nuclear medicine must be carried out in at least two places, and at least one of those places must be equipped with a PET scanner. The other courses cover the basics of human anatomy and physiology, the basics of radiobiology, elements of radiation physics, methods of radiation detection and dosimetry, radiation protection, teleradiotherapy, brachytherapy, non-ionising radiation therapy, bioelectricity and biomagnetism in diagnostics, statistics, elements of medical informatics, and legal and organizational issues. Commencing in 2016, only medical physics specialists will be allowed to perform the advanced nuclear medicine QC procedures. Currently, in nuclear medicine, there are 17 medical physicists with specialization completed or who are enrolled in the specialization program.

Similarly, for those with a master's in chemistry or biology, commencing in 2014, getting a radiopharmacy specialist title becomes possible after completing three years of training and after passing the final national examination. The training includes 167 h of courses for theoretical training and 85 h of practical training. The main goal is a comprehensive education in organizing, performing, and supervising the preparation of radiopharmaceuticals satisfying current requirements. The main courses cover work in aseptic environments, radiation protection during labeling and portioning, radiopharmaceutical preparation and quality control in healthcare units, preparation of radiopharmaceuticals for PET and non-authorised radiopharmaceuticals, and clinical applications of radiopharmaceuticals. Practical training corresponds to all the topics. The other courses cover the basis of pharmacy and legal issues, the basis of radiopharmacy, and the directions of development for radiopharmacy.

### Nuclear medicine specialization for nurses

There is a group of over 350 active electroradiology technologists (approx. 150) and nurses (approx. 200) working in nuclear medicine departments. For 15 years, the Polish Society of Nuclear Medicine has been organizing 1-day courses at least twice a year for the continuing education of these groups.

At present, the specialization program in nuclear medicine for nurses is being developed by the Medical Centre of Postgraduate Education. The training will include 32 h of lectures and 35 h of practical training. The topics will cover radiation protection with elements of radiopharmacology and imaging techniques, medical care of oncology patients during radionuclide diagnostics and therapy, medical care of endocrinology patients during radionuclide diagnostics and therapy, medical care of cardiology patients during radionuclide diagnostics, and medical care during other scintigraphic procedures.

## Continuing education and professional development

Continuing education and professional development are obligatory for all physicians and governed by the Polish Medical Chamber. The general system of credit points in Poland was introduced many years ago; the points are granted by the Polish Medical Chamber and a 4-year settlement period is applied. The responsibility for organizing the upgrade of knowledge training meetings and courses is within the PTMN. The number of credit points achieved is dependent on the program and duration of the course.

The PTMN organizes regular postgraduate training (two to three times a year) for physicians working in nuclear medicine. Additionally, symposia/conferences/workshops/courses are organized each year in collaboration with other scientific societies/organizations like the National Center of Radiation Protection, Polish Society of Endocrinology, Polish Society of Cardiology, Polish Society of Neurology, Polish Society of Oncology, and Polish Society of Radiology. The goal is to update the knowledge of nuclear medicine specialists on radionuclide diagnostics as well as on other diagnostic techniques and clinical applications. Simultaneously, the knowledge about the possibilities of nuclear medicine diagnostic and therapeutic techniques is passed to the doctors of different specializations.

One nuclear medicine department in Poland owns the UEMS/EBNM accreditation for the years 2010–2014. The Polish Ministry of Health, in collaboration with PTMN, has been preparing the program of clinical audits for voluntary nuclear departments. The basic stage of the program, writing all the model procedures, was completed by nuclear medicine experts at the end of 2013.

## International recognition

Three persons have been awarded with the European specialization certificate in radiopharmacy by the Radiopharmacy Committee of EANM.

Numerous members of the Polish nuclear medicine community have contributed to the progress of the specialty via their activities for EANM and UEMS/EBNM:

- Prof. Maciej Gembicki was the Vice-President of the EANM from 1983 to 1986.
- Prof. Gembicki (1975–1984) and Prof. Anna Tarkowska (1988–1993) were members of the Eur J Nucl Med Editorial Board.
- Some specialists have been members of different committees: ● Renata Mikołajczak (Member of the EANM Radiopharmacy Committee 2000–2008, and organizer of the 12th European Symposium on Radiopharmacy and Radiopharmaceuticals, held in Gdańsk, Poland, 2004); ● Anna Płachcińska (Member of the EANM Task Group on Quality Assurance and Standardisation 2000–2001, and EANM Physics Committee 2006–2012); ● Jolanta Kunikowska (Member of the EANM Oncology Committee, at present); ● Daria Handkiewicz-Junak (Secretary of the EANM Radionuclide Therapy Committee, at present).
- Bożena Birkenfeld and Mirosław Dziuk currently hold the position of National Delegates to the UEMS/EBNM; Jolanta Kunikowska and Rafał Czepczyński are delegates to EANM; and Janusz Kapuściński is a member of EANM Radiopharmacy Committee.

Historically, the first European School of Nuclear Medicine had been started by the Polish Society of Nuclear Medicine (1st ESNM Seminar, Warsaw 1995) and three seminars were organized in Poland since then (28th - Warsaw 2004, 36th - Szczecin 2007, and 45th - Warsaw 2010). The local organizers were Dr. Krzysztof Toth, Prof. Leszek Królicki (Warsaw), and Prof. Bożena Birkenfeld (Szczecin).

Beyond the activities for EANM and UEMS/EBNM, Polish professionals have worked for important international organizations connected with nuclear medicine:

- Prof. Julian Liniecki was the expert of the United Nations Scientific Committee on the Effects of Atomic Radiation (UNSCEAR), and from 1964 to 1966 as well as from 1978 to 1986 was the Scientific Secretary of UNSCEAR; he was also a member (now emeritus) from 1969 to present of the International Committee on Radiological Protection (ICRP) Task Group 36 dealing with radiopharmaceutical dosimetry.
- Prof. Maciej Gembicki, in the 1980s, was an expert and a member of the Secretariat of the IAEA in Vienna as well as a WHO expert in charge of providing assistance in organizing centers of nuclear medicine and of promoting research and training in nuclear medicine in many countries of the world.
- Prof. Zbigniew Szybiński, from 1982 to 1994, was an expert of IAEA for the action “Atom for Peace” and organizer of the first nuclear medicine departments in the Sierra Leone capitol and in the University of Malaysia.
- Prof. Eugeniusz Dziuk, from 1990 to 1991, was an IAEA expert with missions to Zimbabwe, Mongolia, Vietnam, and Ethiopia, and the organizer of IAEA Fellowship training for six doctors from Middle Asia at the Department of Nuclear Medicine, Military Institute of Medicine, Warsaw.
- Prof. Barbara Jarząb, since 2001, connects the nuclear medicine with European endocrinology and oncology in the field of thyroid diseases; she is also a member of the European Thyroid Association (ETA), member of the Executive Committee from 2006 to 2009, and was Secretary of ETA-CNR (Cancer Research Network) from 2009 to 2013; and organizer of five international ETA meetings (2009 - Portugal, 2010 - France, 2011 - Poland, 2012 - Italy, and 2013 - the Netherlands).
- Prof. Bożena Birkenfeld, from 2002 to 2005, was an IAEA expert with missions to Tajikistan, Georgia, Kazakhstan, Uzbekistan and was also the organizer of IAEA Fellowship training for five doctors from Middle Asia at the Department of Nuclear Medicine, Pomeranian Medical University in Szczecin. She was also a local organizer of the International Satellite Symposium “Radioisotope Imaging of Infection and Inflammation” (Szczecin-Strzekęcin, 1998) at the 7th Congress of WFNM&B in Berlin and the IAEA Regional Training Course “Scintimammography, lymphoscintigraphy and sentinel lymph node detection” (Szczecin, 2002).
- Prof. Alicja Hubalewska-Dydejczyk, from 2008 to 2010, was the President of the International Research Group in Immuno-Scintigraphy and Therapy (IRIST) and the President of the 19th IRIST Congress held in Kraków, Poland, 2008; she is an Executive Board Member at present.
- Dr. Renata Mikołajczak is an IAEA expert in the area of radiopharmaceuticals.

## Final comments

Educational programs in nuclear medicine in Poland are very comprehensive, covering both diagnostics and current forms of radioisotope therapy. They are aimed not only at physicians specializing in nuclear medicine, but also at other medical professionals employed in radionuclide departments as well as physicians of other specialties.

Postgraduate education and professional development require interpretation of hybrid images and therefore, require skills in evaluating CT and MRI scans. We are working to allow specialists in nuclear medicine to authorize the results of structural examinations.

Our specialists are thoroughly trained in personalized medicine. We find this approach very promising as nuclear medicine offers a wide variety of diagnostic and therapeutic tools that can be applied to patients on an individual basis.

## References

[1] Królicki L, Teresińska A. Achievements of the Polish PET centers. Nucl Med Rev 2012; 15 (Suppl. C): C1-C4.

[2] Prigent A, Huic D, Costa DC (2012). Syllabus for postgraduate specialization in nuclear medicine – 2011/2012 update. Eur J Nucl Med Mol Imaging.

